# Comparative Quantitation of Kokumi γ-Glutamyl Peptides in Spanish Dry-Cured Ham under Salt-Reduced Production

**DOI:** 10.3390/foods12142814

**Published:** 2023-07-24

**Authors:** Alejandro Heres, Qian Li, Fidel Toldrá, René Lametsch, Leticia Mora

**Affiliations:** 1 Instituto de Agroquímica y Tecnología de Alimentos (CSIC), Avenue Agustín Escardino 7, 46980 Paterna, Spain; alejandergo@iata.csic.es (A.H.); ftoldra@iata.csic.es (F.T.); 2 Department of Food Science, Faculty of Science, University of Copenhagen, Rolighedsvej 30, 1958 Copenhagen, Denmark; qianli@food.ku.dk (Q.L.); rla@food.ku.dk (R.L.)

**Keywords:** salt, peptide extraction, kokumi, γ-glutamyl peptides, dry-cured ham, peptidomics

## Abstract

Salting is a crucial step during the production of dry-cured ham and it is not well known whether it has an impact on the generation of taste-active peptides. The present study focused on the quantitation of kokumi γ-glutamyl peptides in low-salted Spanish dry-cured hams with 12 months of processing. By using mass spectrometry, peptides were quantitated from samples obtained after ethanolic deproteinization-based and non-ethanolic deproteinization-based extraction methods. Peptides γ-EA, γ-EE, and γ-EL registered mean values of 0.31, 2.75, and 11.35 µg/g of dry-cured ham, respectively, with no differences observed between both extraction protocols. However, γ-EF, γ-EM, γ-EV, γ-EW, γ-EY, and γ-EVG presented significantly (*p* < 0.05) higher concentrations in the ethanolic deproteinized samples showing values of 5.58, 4.13, 13.90, 0.77, 3.71, and 0.11 µg/g of dry-cured ham, respectively. These outcomes reflect the importance of protocols for the extraction of peptides to achieve the most feasible results. In addition, potential precursors for the formation of γ-glutamyl peptides are generated during dry-curing under salt restriction. The kokumi activity of these γ-glutamyl peptides could enhance the sensory attributes countering the taste deficiencies caused by the salt restriction.

## 1. Introduction

Dry-cured ham is a world-widely consumed product with special relevance in the Mediterranean area. Such popularity is due to its sensory properties, which are a consequence of the biochemical reactions occurred during its processing. Among them, lipolysis and proteolysis are the principal routes leading to the generation of taste-active compounds, from which short peptides play special relevance [1].

In relation to taste, salting constitutes a crucial step in the elaboration of dry-cured ham. However, while reducing the content of salt could improve the conception of this product, issues related to food safety and a potential sensory disruption should be consequently addressed. Salt is known to promote oxidative pathways [2], and exerts inhibitory effects on proteolytic enzymes [3]. Several comparative attempts have been conducted to determine the sensory consequences and the peptidase activity modulation of low-salted dry-cured products [4,5]. Notwithstanding, little research has been focused on the influence of lowering the amount of salt on the generation of taste-active peptides, which could have a key impact on the final taste [6]. γ-Glutamyl peptides are small molecular *peptides* that greatly influence the perception of the kokumi taste [7]. Kokumi γ-glutamyl peptides might be the responsible molecules of the attribute “brothy” previously identified in fractions derived from gel fractionation of Spanish dry-cured ham peptide extracts [8]. These peptides are synthesized by enzymes that participate in the glutathione (GSH) cycle, such as γ-glutamyl transpeptidase, whose activity has been identified in pork [9]. Despite the proteolysis that occurs in dry-cured ham’s processing being a consequence mainly of the action of endogenous muscle proteases, and while the microbial activity is not significant to the generation of peptides [10], a high diversity of species that exert γ-glutamyl transpeptidase activity can colonize the ham pieces [11,12,13]. γ-Glutamyl transferase, γ-glutamyl transpeptidase, γ-EC synthetase, and GSH synthetase are enzymes that can produce kokumi peptides during fermentations [11]. 

Their generation could be conditioned by the γ-glutamyl transpeptidase activity and the availability of substrates (free amino acids), which could be affected by the concentration of salt [10,14,15]. Given the relevance of these kokumi peptides in sensory enhancement [16] and the role of salt on the development of the typical dry-cured ham taste [17], the analysis of γ-glutamyl peptides in dry-cured ham under salt restriction could provide better knowledge for the elaboration of healthier and tastier products.

For this purpose, the analysis of short peptides by mass spectrometry (MS) requires dealing with several challenges [18]. The first one is the method for the extraction of the aqueous peptide fraction from dry-cured ham samples [19,20,21,22], and special consideration should be made to minimize peptide losses during deproteinization. The ethanolic deproteinization (ED)-based method described in Mora et al. (2009) [23], has allowed to identify and quantitate a large number of α-dipeptides in Spanish dry-cured ham elaborated with lower content of salt [6] aside from numerous longer α-peptides [24]. In contrast, a former investigation [21] was successful in quantitating γ-EF, γ-EI, γ-EL, and γ-EY in traditional Parma dry-cured ham extracts obtained using a protocol that did not include a deproteinization step (non-ethanolic deproteinization, NED). It has been reported that nondenaturing methods for the extraction of peptides provide a major extraction of high molecular weight proteins while denaturing conditions benefit the collection of fragments with low molecular weight [25].

In the present study, it was decided to compare the impact of both extraction protocols, the ED and the NED-based methodology, on the quantitation of γ-glutamyl peptides in the low-salted 12-month-aged Spanish dry-cured ham. In this sense, no study following both ED- and NED-based extractions has been conducted focusing on the generation of γ-glutamyl peptides as a function of the reduction of salt in the dry-curing of ham. The comparison between methods would reveal proven evidence about the best protocol for the extraction of these compounds, to obtain the most feasible quantitative results; besides which no quantitation of γ-glutamyl peptides in low-salted Spanish dry-cured ham has been carried yet and would demonstrate a γ-glutamyl transferase activity that could be crucial for the development of taste. 

## 2. Materials and Methods

### 2.1. Chemicals and Reagents

Commercial γ-glutamyl dipeptides with kokumi properties [11,26] γ-EA and γ-ECG (GSH) were acquired from Sigma-Aldrich (Steinheim, Germany), γ-EC from APExBIO (Huston, TX, USA), and γ-EVG from Fujifilm Wako Chemicals (Tokyo, Japan). Finally, γ-EE, γ-EF, γ-EG, γ-EL, γ-EM, γ-EQ, γ-EV, γ-EW, and γ-EY were purchased from Bachem (Weil am Rhein, Germany). Hydrochloric acid and ethanol (analytical grade) were purchased from Scharlab, S.L. (Barcelona, Spain). The remaining reagents were of analytical grade, acquired from Sigma-Aldrich (Steinheim, Germany). 

### 2.2. Total Peptide Extraction and Ultrafiltration

Four dry-cured hams from pigs of industrial genotypes *Landrace x Large White* were processed under a reduced amount of sodium chloride (final salt content of 3.3%, *w*/*w*; traditional dry-cured hams have between 5 and 6% of salt) until 12 months of curing in the factory Incarlopsa (Tarancón, Spain) was complete. The manufacturing of the dry-cured hams consisted of several stages, namely: pre-salting, in which hams are treated with a mixture of curing ingredients; salting, in which hams are covered in salt and placed in salting chambers for 11–12 days at a temperature of 4 °C and a relative humidity of 85–90%; post-salting, which consists of brushing the hams and they are kept below 4 °C, and the relative humidity ranges from 75% to 85% for 50 days; and finally, ripening, in which hams are transferred to air-conditioning chambers and undergo specific cycles of time, temperature, and relative humidity [27]. 

Peptides were extracted from *Biceps femoris* muscles. A total of 5 g was collected from samples to be processed with each extraction protocol. The resulting extracts from both protocols were used in the determination of amino acids and in the quantitation of the γ-glutamyl peptides. 

#### 2.2.1. Ethanolic Deproteinization-Based Method

According to the methodology described by Mora et al. [23], samples were homogenized for 8 min under 4 °C with 20 mL 0.01 N HCl in a stomacher (IUL Instruments, Barcelona, Spain). After centrifugation for 20 min at 12,000× *g* and under 4 °C, they were filtered through glass wool. Protein precipitation was triggered by diluting the filtrate 1:4 in ethanol and keeping the mixture at 4 °C for 20 h. The resulting suspension was centrifuged at the same former conditions and evaporated under vacuum in a rotary evaporator to be lyophilized next in a freeze dryer (SCANVAC CoolSafe, Labogene APS, Lynge, Denmark). Dry matter was dissolved in 22.5 mL of 0.1% formic acid aqueous solution and centrifuged at 20,000 rpm for 20 min to be then filtrated with a 0.45 µm nylon filter (Teknokroma, Barcelona, Spain). A volume of 500 µL of that filtrate was filtered again with a 10 KDa cut-off filter (UFC501096, Merck Millipore, Billerica, MA, USA) under 15,000 rpm for 20 min at 4 °C and lyophilized in a previously weighed tube. Triplicates from each sample were taken at this second filtration.

#### 2.2.2. Non-Ethanolic Deproteinization-Based Method

Considering the methodology from Paolella et al. (2018) [21], a second extraction protocol was conducted with several modifications. An amount of minced 5 g was homogenized with 45 mL of 0.1 N HCl in an Ultra Turrax for 1 min. Once centrifuged at 12,000× *g* for 20 min at 4 °C, samples were filtrated with glass wool, and 4 mL of the filtrate was vacuum evaporated by a rotary evaporator (G3 Heidolph, Schwabach, Germany). The residue was dissolved in 2 mL of bi-distilled water and lyophilized to be then resuspended in 2 mL of 0.1% formic acid aqueous solution. After this, samples were centrifuged at 20,000 rpm for 20 min under 4 °C and filtrated with a 0.45 µm nylon filter. Finally, a volume of 150 µL of that filtrate was filtered again with a 10 kDa cut-off filter (UFC501096, Merck Millipore, Billerica, MA, USA) under 15,000 rpm for 20 min at 4 °C and lyophilized (SCANVAC CoolSafe, Labogene APS, Lynge, Denmark) in a previously weighed tube. Triplicates from the same sample were collected in this step. 

### 2.3. Amino Acids Determination

A similar procedure as followed by Flores et al. (1997) [17] was performed, with several modifications. After filtration at 0.45 µm with nylon filters of deproteinized samples, 150 μL of each one was derivatized. Amino acid chromatographic separation was achieved by using a reversed-phase high-performance liquid chromatography (RP-HPLC) system (Series 1100; Agilent, Santa Clara, CA, USA) counting with a Waters Nova Pack^®^ C18 column (3.9 × 300 mm; Waters Corporation, Milford, MA, USA). A gradient was generated between solvent A (70 mM sodium acetate pH 6.55 and 2.5 acetonitrile) and solvent B (4.5:4.0:1.5 of acetonitrile, water, and methanol, respectively). Temperature was set at 52 °C and the amino acids were detected at 254 nm. The concentrations were predicted by introducing the peak areas into standard curves. 

### 2.4. Tageted Quantitative Analysis of γ-Glutamyl Peptides

The identification and quantitation of the kokumi γ-glutamyl peptides were conducted based on the methodology by Li et al. (2020) [16]. The MS system consisted of a Dionex Ultimate 3000 Ultra High Performance Liquid Chromatography (UHPLC) device (Thermo Fisher Scientific, Hvidovre, Denmark) connected to a Q Exactive Orbitrap mass spectrometer (Thermo Fisher Scientific, Roskilde, Denmark). The analysis was performed with a positive electrospray ionization source (ESI) and under parallel reaction monitoring (PRM) mode. 

Prior to the analysis, ultrafiltered peptide extracts were dissolved in 150 µL of bidistilled water plus 1 µL of trifluoroacetic acid. Subsequently, an aliquot of 10 μL was injected onto a C18 column (BioZen, 1.7 μm Peptide XB-C18, 150 × 2.1 mm^2^, Phenomenex, Værløse, Denmark). Mobile phase A consisted of 0.1% formic acid aqueous solution and mobile phase B consisted of 0.1% formic acid in 80% acetonitrile. Elution occurred at a flow rate of 0.25 mL/min, under the following gradient: 0–5.0 min 100% A, 5.0–20.0 min 0–30% B, 20.0–20.5 min 30–100% B, 20.5–25.0 min 100% B, 25.0–26.0 min 0–100% A, and 26.0–30.0 min 100% A.

The scan events included an MS1 full scan with a resolution of 17,500 from 50 to 750 *m*/*z*, followed by PRM scans of the precursors included in the list with a resolution of 17,500, an AGC target of 1e, a maximum IT of 64 ms, and an isolation window of 1.0 *m*/*z*. The quantitation was calculated after checking the retention times and fragmentation patterns published in Li et al. (2020) [16], by interpolation of the peak area of each peptide in the standard equation curve with Xcalibur software 4.1. 

To discard their α-bound homologs, considering that elimination of ammonia is usually characteristic of protonated dipeptides with a γ-linkage, the ions [M–NH_3_ + H]^+^ of reference compounds were chosen for calibration curves [16,19,28] (M represents the parental ion).

Concentrations of γ-glutamyl peptides in samples are expressed as means of 4 biological replicates ± standard deviation. Statistical analysis for the concentrations was executed considering a significance level of 0.05 and involved performing one-way analysis of variance (ANOVA) and Tukey’s all-pair comparisons as post hoc, by using the software RStudio v1.4.1103 (Boston, MA, USA). The amino acid principal component analysis (PCA) was calculated with SIMCA v13.0 (Umetrics AB, Goettingen, Sweden), under a 95% of confidence.

## 3. Results

### 3.1. Amino Acids Determination

In general, larger amounts of amino acids were collected from samples extracted with the NED-based method. As presented in Table 1, most of them showed significant differences (*p*-value < 0.05) when comparing both methods. On average, approximately 1.5 times more amino acids were collected using the ED-based method than using the ED-based method.

More precisely, Gln was not measurable in ED-based method-derived samples, despite a low amount recovered from NED-based method samples (53.33 µg/g dry-cured ham). The amino acids Lys, Glu, Leu, and Ala resulted as being the most abundant, with NED-based extraction-derived concentration values reaching 11,910.47, 5502.06, 5258.94, and 3855.85 µg/g dry-cured ham, respectively; while low amounts were obtained for Tyr, Trp, Asn, and Gln, registering 597.08, 411.68, 304.25, and 53.33 µg/g dry-cured ham, respectively, from the NED-based extracted samples. 

### 3.2. γ-Glutamyl Peptides-Targeted Quantitative Analysis

From a total of 13 γ-glutamyl peptide standards (γ-EA, γ-EC, γ-EE, γ-EF, γ-EG, γ-EL, γ-EM, γ-EQ, γ-EV, γ-EW, γ-EY, γ-EVG, and GSH), 8 γ-glutamyl dipeptides (γ-EA, γ-EE, γ-EF, γ-EL, γ-EM, γ-EV, γ-EW, and γ-EY) and a γ-glutamyl tripeptide (γ-EVG) were able to be quantitated in low-salted samples from dry-cured hams at 12 months of dry-curing. In contrast, γ-EC, γ-EG, γ-EQ, and GSH were absent, or their concentrations were below the limit of quantitation.

**Figure 1:**
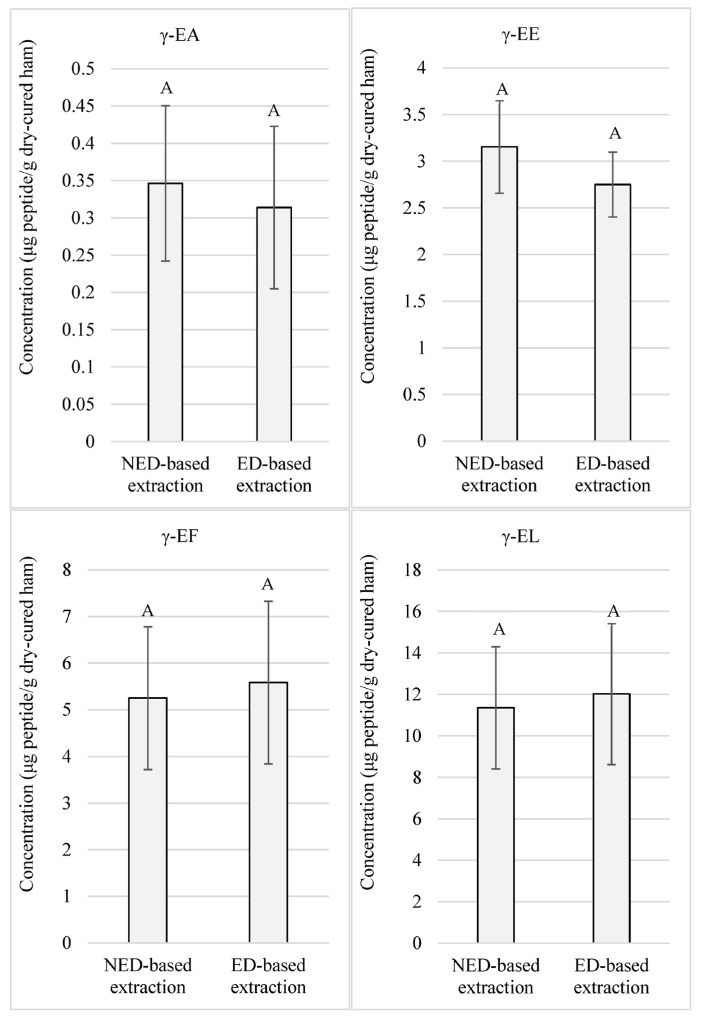
Quantitated γ-glutamyl peptides in low-salted 12-month-aged Spanish dry-cured hams according to 2 extraction protocols: NED-based extraction refers to the extraction protocol that does not require ethanolic deproteinization. ED-based extraction refers to the extraction protocol that uses ethanolic deproteinization. Same letters indicate non-significant differences.

It was observed that concentrations of most peptides reached levels in the order of microgram per gram of dry-cured ham. Comparing the concentrations between both extraction methods, non-significant differences (*p*-value > 0.05) were recorded for γ-EA, γ-EE, γ-EF, and γ-EL, as presented in Figure 1. These peptides reached values above 0.31, 2.75, 5.25, and 11.35 μg of peptide/g of low-salted dry-cured ham, respectively.

**Figure 2:**
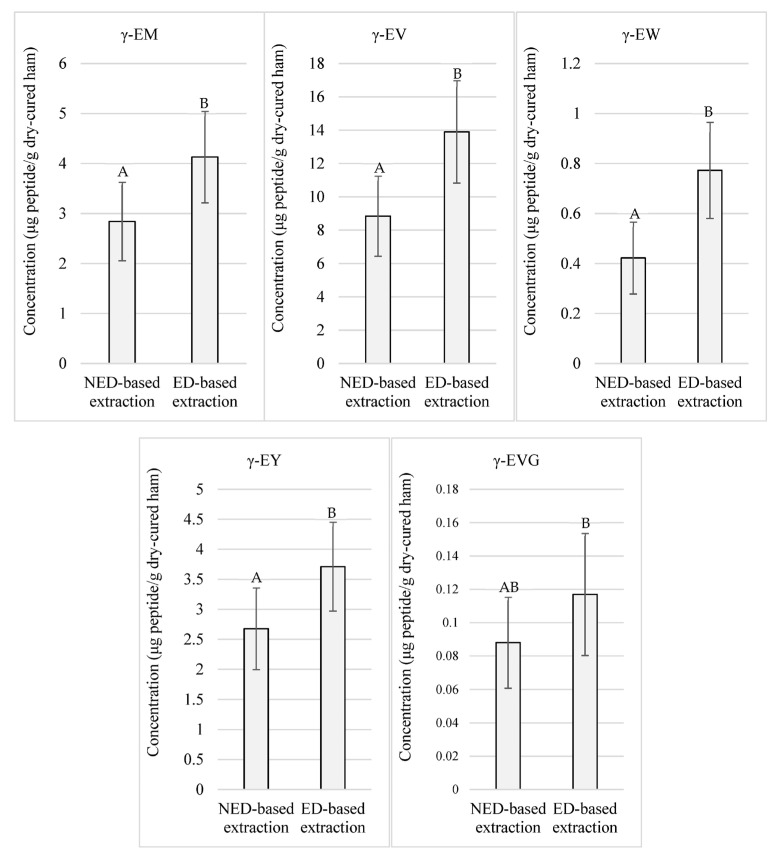
Quantitated γ-glutamyl peptides in low-salted 12-month-aged Spanish dry-cured hams according to two extraction protocols: NED-based extraction refers to the extraction protocol that does not require ethanolic deproteinization. ED-based extraction refers to the extraction protocol that uses ethanolic deproteinization. Same letters indicate non-significante differences.

Otherwise, as exposed in Figure 2, significant (*p* < 0.05) higher concentrations were registered for γ-EM, γ-EV, γ-EW, γ-EY, and γ-EVG coming from the peptide extracts obtained through the ED-based methodology, taking values above 4.13, 13.90, 0.77, 3.71, and 0.11 μg of peptide/g of low-salted dry-cured ham, respectively, on average in those corresponding samples. 

**Figure 3:**
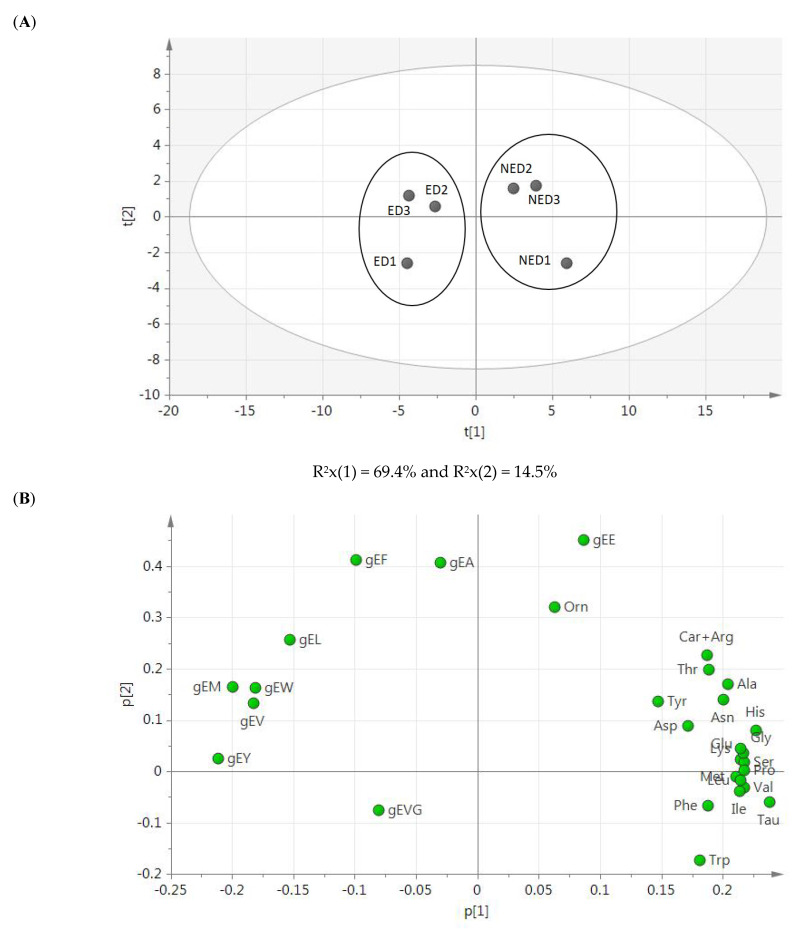
PCA results. (**A**) Score plot of amino acids and γ-glutamyl peptides identified in low-salted 12-month-aged Spanish dry-cured hams, to assess the variance among the compounds generated using two different extraction protocols (*n* = 3); and (**B**) loading plot showing the peptides affecting the score plot distribution. ED indicates ethanolic deproteinization-derived samples and NED is for non-ethanolic deproteinization-derived samples.

The PCA shown in Figure 3 revealed the main differences for γ-glutamyl peptides’ data, especially in the group of samples from the ED-based protocol (Figure 3A). Additionally, while the first component had large positive associations with amino acids and except for γ-EE, the second component had large positive associations with the γ-glutamyl peptides, except for γ-EVG, whose association was negative (Figure 3B).

## 4. Discussion

Previous scientific reports have documented the progressive accumulation of peptides during the dry-cured processing of ham as a consequence of the proteolytic action of muscle peptidases [24]. However, it is also well known that environmental conditions have a modulatory influence on enzymatic activities [3]. Thus, a better comprehension of the role of salt on the generation of peptides with taste-active properties would be of high relevance to producing healthier and regular batches with the best sensory properties. 

High-ionic strength conditions make the proteins lose their conformation and be more susceptible to free radicals-related reactions. Therefore, the reduction of salt might lead to the amelioration of oxidative processes during the dry-curing period. Post-translational peptide modifications, including oxidation, are part of the biochemical processes which determine the sensory properties of these products. However, the excess of these biochemical routes can lead to unpleasant flavors, which can be due to the degradation of key taste-active peptides [29].

For these reasons, peptidomic approaches are essential for the study of the generation of small peptides, although several challenges must be addressed in order to optimize the determination [18]. The suppression of mass spectrometry signal by salt [30] should be confronted by a convenient peptide extraction method, which should ensure the maximum recovery of the peptides contained in the sample. 

### 4.1. Amino Acids Determination

Overall, the former research results provide evidence that cutting down the amount of salt employed during dry-curing alters the concentration of amino acid-derived compounds [4,5,6,31,32]. Based on this premise, the amino acid content in low-salted dry-cured hams was accomplished in this research. 

Regarding the present study, higher amounts of amino acids were collected in NED-based method-derived samples, which suggests that the organic solvent used for the deproteinization followed by incubation for 12 h and centrifugation afterward produces amino acid losses in the ED-based protocol. Amino acids are relatively high polar compounds, and therefore, they might not be well dissolved in the organic solvent and thus would remain in the insoluble fraction during centrifugation. 

### 4.2. γ-Glutamyl Peptides-Targeted Quantitative Analysis

The development and optimization of mass-spectrometry-based techniques have allowed to identify and quantitate many short peptides in several types of dry-cured hams [20,33,34,35,36]. However, almost all the identified peptides are formed by α-type linkages, and few publications have disclosed the identification and quantification of γ-glutamyl peptides in dry-cured hams despite their potential relevance to the development of taste. Peptides γ-EF, γ-EI, and γ-EL were identified and semiquantitated in 450, 570 and 690 days-elaborated Parma hams [37], and in a subsequent study, γ-EF, γ-EI, and γ-EL were identified in 24-month-aged Parma dry-cured hams, with concentrations reaching approximately 15, 25, and 45 µg/g dry-cured ham, respectively [21]. γ-EYwas also detected in that last publication at lower concentrations [21]. In another study, apart from the identified peptides γ-EF and γ-EY, γ-EW was relatively quantified in 14-, 22- and 34-month-processed dry-cured hams [19]. 

This research successfully identified 8 γ-glutamyl dipeptides, γ-EA, γ-EE, γ-EF, γ-EL, γ-EM, γ-EV, γ-EW, and γ-EY; and a γ-glutamyl tripeptide, γ-EVG; and also, their absolute quantitation was possible. However, other peptides like γ-EC, γ-EG, γ-EQ, and GSH were absent or their concentrations were below the limit of quantitation.

Differences regarding both peptide extraction protocols include the first homogenization in 45 mL of 0.1 N HCl in Ultra Turrax for 1 min in the NED-based protocol or in 20 mL 0.01 N HCl in stomacher for 8 min in the ED-based protocol. The latter operation seems to be more respectful of the integrity of the proteins. After filtration of the homogenates with glass wool in the NED-based protocol, 4 mL of the filtrate was dried using a rotary evaporator; while in the ED-based protocol, this was conducted with all the filtrate volume for ethanol removal after 20 h of deproteinization. This step might be the greatest cause of peptide loss when drying 4 mL instead of the whole sample. Next, the dried matter was dissolved in 2 mL of 0.1% formic acid aqueous solution in the NED-based method, whereas in the ED-based method, the samples were dissolved in 22.5 mL 0.1% formic acid aqueous solution to compensate for the concentration due to the different HCl aqueous solution volumes used for the homogenization. Moreover, while in the NED-based method, 150 μL of the sample was directly ultrafiltered, in the other method, samples were firstly filtrated with a 0.45 µm filter and then, 500 μL of this filtrate was ultrafiltered [21,23].

As has been already mentioned, peptides γ-EM, γ-EV, γ-EW, γ-EY, and γ-EVG presented higher recovery yields with the ED-based protocol, appearing to be a better option to analyze γ-glutamyl peptides in dry-cured ham. 

In previous studies, the inhibitory role of salt on the activity of peptidases has been described [14], so a decrease in salt concentration could result in a larger amount of released peptides and free amino acids. Here, a high amount of amino acids was perceived, which suggests intense proteolysis in samples. 

In a previous study, α-bound dipeptides DA, DG, EE, ES, GA, PA, and VG have been quantitated in traditional and low-salted Spanish dry-cured hams. The statistical analysis revealed that the concentrations of peptides DA, PA, and VG, were significantly (*p* < 0.05) higher in traditional-salted dry-cured hams [35] and this fact might be attributed to the potential increase in aminopeptidase’s activity in the presence of lower salt amounts [14]. In addition, the sweet and potential antihypertensive dry-cured ham-derived dipeptide AA exhibited no statistical differences between traditional-salted and low-salted samples [38]. 

On the other hand, γ-EC and GSH participate in oxidative metabolism, and, provided that salt promotes oxidative reactions, an increase in the concentration of these peptides was expected in low-salted hams due to a less hypothetical consumption as a consequence of oxidative stress [39]. Thus, as salt has an inhibitory effect on peptidases [3] and γ-glutamyl peptides are resistant to the proteolytic action [37], the reduction of salt might unveil other reactions by which both peptides would be consumed, or definitely, not generated during processing. In addition to this, salt can exert different modulatory effects on γ-glutamyl transpeptidases. *Bacillus amyloliquefaciens* S0904 γ-glutamyl transpeptidase has been reported to be inhibited by NaCl [40], while in contrast, purified *Escherichia coli* K-12 strain CY6 (pCY2/SH641) γ-glutamyl transpeptidation has been observed to result enhanced by NaCl in vitro [41]. The absence of data of γ-EC and GSH in low-salted dry-cured ham samples might be due to other routes uninhibited, or by a decrease in the γ-glutamyl transpeptidation activity, due to the reduction of salt. Considering that γ-EC and GSH are known to function as substrates for γ-glutamyl transferase [16], these peptides might also be used by γ-glutamyl transpeptidases at a higher rate in the presence of a less salted environment, favoring the generation of other γ-glutamyl peptides. Thus, the high concentrations of γ-glutamyl peptides observed in this study using any of both extraction protocols (Figure 1 and Figure 2) might be explained because of the enhancement of the γ-glutamyl transpeptidase activity triggered by the reduction of salt. 

The aromatic C-terminal γ-glutamyl dipeptides γ-EF, γ-EW, and γ-EY reached higher concentrations in low-salted dry-cured hams, which confirms that less expected oxidative action due to the reduction of salt would tend to consume less aromatic-consisting peptides. For the case of γ-EVG, the generation of VG in low-salted dry-cured ham [6] might promote the production of the γ-glutamyl peptide. 

Further, the availability of free amino acids probably would have an influence on the generation of γ-glutamyl peptides. It is known that Gln amino acid constitutes a substrate for γ-glutamyl transpeptidation [16]. Due to the wide range of different γ-glutamyl peptides identified in this study, its consumption during γ-glutamylation might be the reason they were almost not detected using both extraction methodologies. It might occur that those free amino acids at higher concentrations could be used as substrates for γ-glutamylation. In this sense, the C-terminal residues of γ-EA and γ-EL were the most concentrated in their free form. Notwithstanding, the proportionality may not be fulfilled, and it might consist of a matter of enzymatic affinity [42] since Tyr was one of those that were present in the lowest concentration and γ-EY seemed to be concentrated in moderate amounts. This is interesting as it has been published that Val, Met, Phe, Trp, and Tyr display the lowest K_m_ values [11]. In the case of Trp, present at low concentrations, γ-EW was also one of the scarcest peptides in its γ-glutamylated form. Another example can be that of the abundant Glu and the relatively moderated concentration of γ-EE. 

Supporting results were extracted from the PCA plot, from which it can be deduced that the main differences between the samples obtained by the two different peptide extraction protocols are due to the concentration of the γ-glutamyl peptides, especially that of γ-EE and in comparison, with the rest of them. It seemed that the quantitation of amino acids was not relevant for the differentiation of both samples despite significantly (*p*-value < 0.05) greater concentrations registered in those from the NED-based protocol. 

Overall, based on the data from the present research it has been proved the generation of γ-glutamyl peptides along the dry-curing process with a reduction of the amount of salt in the salting step. Due to the synergistic effect of the γ-glutamyl peptides [16], the detected concentrations of the γ-glutamyl peptides studied in this report might exceed the threshold to sense the kokumi effect, providing flavor-improving effects that compensate for the sensory detriments resulting from the reduction of salt in the processing [5]. 

Future research might involve the reduction of salt in different percentages to assess the formation of γ-glutamyl peptides and the characterization of the γ-glutamyl transferase activity along the processing of dry-cured ham.

## 5. Conclusions

Using an MS spectrometry technique based on reversed-phase high-performance liquid chromatography coupled to a Q Exactive orbitrap mass spectrometer, γ-EA, γ-EE, γ-EF, γ-EL, γ-EM, γ-EV, γ-EW, γ-EY, and γ-EVG were quantitated in samples derived from 12-month-aged dry-cured hams elaborated with a lower content of salt. Two peptide extraction protocols, previously used in other studies, were used to consider the most recommendable for the quantitative analysis of these peptides. 

The peptide extraction methodology based on an ethanolic deproteinization resulted in the recovery of higher amounts of peptides in comparison with the non-ethanolic deproteinization protocol, evidencing the relevance of sample preparation prior to the mass spectrometry analysis, and being the most recommendable protocol for extraction of γ-glutamyl peptides. 

The free amino acids identified in the low-salted samples would serve as precursors for γ-glutamylation. The kokumi activity of the resulting short γ-glutamyl peptides found in dry-cured ham elaborated with a reduced content of salt might counteract the sensory deficiencies produced by the salt restriction and improve the taste of the final product. 

## Figures and Tables

**Table 1:** Content of free amino acids (µg amino acid/g dry-cured ham) in low-salted 12-month-aged Spanish dry-cured hams obtained by two different peptide extraction protocols.

	ED-Based Method		NED-Based Method	
	Average	SD	Average	SD
Asp	1687.91 ^a^	211.78	2377.84 ^b^	329.34
Glu	3347.55 ^a^	425.62	5502.06 ^b^	567.84
Ser	1569.98 ^a^	188.38	2893.25 ^b^	380.54
Asn	149.88 ^a^	63.73	304.25 ^a^	105.75
Gly	1342.80 ^a^	183.80	2439.31 ^b^	309.56
Gln	NQ	NQ	53.33	3.48
His	996.27 ^a^	96.61	1427.74 ^b^	597.85
Thr	1573.51 ^a^	199.89	2562.46 ^b^	262.47
Ala	2668.40 ^a^	369.60	3855.85 ^b^	290.97
Pro	1840.77 ^a^	96.24	3020.00 ^b^	360.21
Tyr	371.89 ^a^	28.64	597.08 ^b^	133.21
Val	2319.63 ^a^	258.43	4030.81 ^b^	661.33
Met	970.91 ^a^	146.31	1593.6 ^b^	294.79
Ile	1792.02 ^a^	286.36	3115.8 ^b^	602.42
Leu	3028.03 ^a^	517.82	5258.94 ^b^	945.43
Phe	1364.47 ^a^	54.12	2094.46 ^a^	975.06
Trp	328.76 ^a^	57.85	411.68 ^a^	171.72
Lys	7514.67 ^a^	1514.19	11,910.47 ^b^	1196.10

Amino acids are given in a three-letter code. Different letters in superscript format indicate significant (*p* < 0.05) differences between concentration values from the two different extraction protocols: ethanolic deproteinization-based method (ED-based method) and non-ethanolic deproteinization-based method (NED-based method).

## Data Availability

The data used to support the findings of this study can be made available by the corresponding author upon request.

## Abbreviations

A, Alanine; C, Cysteine; D, Aspartate; E, Glutamate; F, Fenilalanine; G, glycine; H, Histidine; I, Isoleucine; K, Lysine; L, Leucine; M, Methionine; N, Asparagine; P, Proline; Q, Glutamine; R, Arginine; S, Serine; T, Threonine; V, Valine; W, Tryptophan; Y, Tyrosine; MS, mass spectrometry; ED, ethanolic deproteinization; NED, non-ethanolic deproteinization, PRM, parallel reaction monitoring; RP-HPLC, reversed-phase high-performance liquid chromatography; UHPLC, ultra-high-performance liquid chromatography; ESI, electrospray ionization source; ANOVA, one-way analysis of variance; PCA, principal component analysis.

## References

[1] Toldrá F., Flores M. (1998). The Role of Muscle Proteases and Lipases in Flavor Development during the Processing of Dry-Cured Ham. Crit. Rev. Food Sci. Nutr..

[2] Heres A., Yokoyama I., Gallego M., Toldrá F., Arihara K., Mora L. (2022). Impact of Oxidation on the Cardioprotective Properties of the Bioactive Dipeptide AW in Dry-Cured Ham. Food Res. Int..

[3] Sentandreu M.A., Toldrá F. (2001). Dipeptidyl Peptidase Activities along the Processing of Serrano Dry-Cured Ham. Eur. Food Res. Technol..

[4] Armenteros M., Aristoy M.C., Barat J.M., Toldrá F. (2009). Biochemical Changes in Dry-Cured Loins Salted with Partial Replacements of NaCl by KCl. Food Chem..

[5] Armenteros M., Aristoy M.-C., Barat J.M., Toldrá F. (2009). Biochemical and Sensory Properties of Dry-Cured Loins as Affected by Partial Replacement of Sodium by Potassium, Calcium, and Magnesium. J. Agric. Food Chem..

[6] Heres A., Gallego M., Mora L., Toldrá F. (2022). Identification and Quantitation of Bioactive and Taste-Related Dipeptides in Low-Salt Dry-Cured Ham. Int. J. Mol. Sci..

[7] Wang H., Suo R., Liu X., Wang Y., Sun J., Liu Y., Wang W., Wang J. (2022). Kokumi γ-Glutamyl Peptides: Some Insight into Their Evaluation and Detection, Biosynthetic Pathways, Contribution and Changes in Food Processing. Food Chem. Adv..

[8] Sentandreu M.Á., Stoeva S., Aristoy M.C.C., Laib K., Voelter W., Toldra E., Toldrá F., Toldra E. (2003). Identification of Small Peptides Generated in Spanish Dry-Cured Ham. J. Food Sci..

[9] Rico A.G., Braun J.P., Benard P., Thouvenot J.P. (1977). Tissue and Blood Gamma-Glutamyl Transferase Distribution in the Pig. Res. Vet. Sci..

[10] Toldrá F., Flores M., Sanz Y. (1997). Dry-Cured Ham Flavour: Enzymatic Generation and Process Influence. Food Chem..

[11] Yang J., Bai W., Zeng X., Cui C. (2019). Gamma Glutamyl Peptides: The Food Source, Enzymatic Synthesis, Kokumi-Active and the Potential Functional Properties—A Review. Trends Food Sci. Technol..

[12] Martínez-Onandi N., Sánchez C., Nuñez M., Picon A. (2019). Microbiota of Iberian Dry-Cured Ham as Influenced by Chemical Composition, High Pressure Processing and Prolonged Refrigerated Storage. Food Microbiol..

[13] Blesa E., Aliño M., Barat J.M., Grau R., Toldrá F., Pagán M.J. (2008). Microbiology and Physico-Chemical Changes of Dry-Cured Ham during the Post-Salting Stage as Affected by Partial Replacement of NaCl by Other Salts. Meat Sci..

[14] Toldrá F., Cerveró M.C., Part C. (1993). Porcine Aminopeptidase Activity as Affected by Curing Agents. J. Food Sci..

[15] Shuai Y., Zhang T., Mu W., Jiang B. (2011). Purification and Characterization of γ-Glutamyltranspeptidase from *Bacillus subtilis* SK11.004. J. Agric. Food Chem..

[16] Li Q., Liu J., De Gobba C., Zhang L., Bredie W.L.P., Lametsch R. (2020). Production of Taste Enhancers from Protein Hydrolysates of Porcine Hemoglobin and Meat Using *Bacillus amyloliquefaciens* γ-Glutamyltranspeptidase. J. Agric. Food Chem..

[17] Flores M., Aristoy M.-C., Spanier A.M., Toldrá F. (1997). Non-Volatile Components Effects on Quality of “Serrano” Dry-Cured Ham as Related to Processing Time. J. Food Sci..

[18] Mora L., Gallego M., Reig M., Toldrá F. (2017). Challenges in the Quantitation of Naturally Generated Bioactive Peptides in Processed Meats. Trends Food Sci. Technol..

[19] Cerrato A., Aita S.E., Capriotti A.L., Cavaliere C., Montone A.M.I., Montone C.M., Laganà A. (2022). Investigating the Short Peptidome Profile of Italian Dry-Cured Ham at Different Processing Times by High-Resolution Mass Spectrometry and Chemometrics. Int. J. Mol. Sci..

[20] Degnes K.F., Kvitvang H.F.N., Haslene-Hox H., Aasen I.M. (2017). Changes in the Profiles of Metabolites Originating from Protein Degradation During Ripening of Dry Cured Ham. Food Bioprocess Technol..

[21] Paolella S., Prandi B., Falavigna C., Buhler S., Dossena A., Sforza S., Galaverna G. (2018). Occurrence of Non-Proteolytic Amino Acyl Derivatives in Dry-Cured Ham. Food Res. Int..

[22] Mora L., Gallego M., Toldrá F. (2019). Degradation of Myosin Heavy Chain and Its Potential as a Source of Natural Bioactive Peptides in Dry-Cured Ham. Food Biosci..

[23] Mora L., Sentandreu M.Á., Koistinen K.M., Fraser P.D., Toldrá F., Bramley P.M. (2009). Naturally Generated Small Peptides Derived from Myofibrillar Proteins in Serrano Dry-Cured Ham. J. Agric. Food Chem..

[24] Toldrá F., Gallego M., Reig M., Aristoy M.-C., Mora L. (2020). Bioactive Peptides Generated in the Processing of Dry-Cured Ham. Food Chem..

[25] Malva A.D., Albenzio M., Santillo A., Russo D., Figliola L., Caroprese M., Marino R. (2018). Methods for Extraction of Muscle Proteins from Meat and Fish Using Denaturing and Nondenaturing Solutions. J. Food Qual..

[26] Li Q., Zhang L., Lametsch R. (2022). Current Progress in Kokumi-Active Peptides, Evaluation and Preparation Methods: A Review. Crit. Rev. Food Sci. Nutr..

[27] Mora L., Sentandreu M.Á., Fraser P.D., Toldrá F., Bramley P.M. (2009). Oligopeptides Arising from the Degradation of Creatine Kinase in Spanish Dry-Cured Ham. J. Agric. Food Chem..

[28] Harrison A.G. (2004). Characterization of α- Andγ-Glutamyl Dipeptides by Negative Ion Collision-Induced Dissociation. J. Mass Spectrom..

[29] Soladoye O.P., Juárez M.L., Aalhus J.L., Shand P., Estévez M. (2015). Protein Oxidation in Processed Meat: Mechanisms and Potential Implications on Human Health. Compr. Rev. Food Sci. Food Saf..

[30] Metwally H., McAllister R.G., Konermann L. (2015). Exploring the Mechanism of Salt-Induced Signal Suppression in Protein Electrospray Mass Spectrometry Using Experiments and Molecular Dynamics Simulations. Anal. Chem..

[31] Lorenzo J.M., Cittadini A., Bermúdez R., Munekata P.E., Domínguez R. (2015). Influence of Partial Replacement of NaCl with KCl, CaCl_2_ and MgCl_2_ on Proteolysis, Lipolysis and Sensory Properties during the Manufacture of Dry-Cured Lacón. Food Control.

[32] Muñoz-Rosique B., Salazar E., Tapiador J., Peinado B., Tejada L. (2022). Effect of Salt Reduction on the Quality of Boneless Dry-Cured Ham from Iberian and White Commercially Crossed Pigs. Foods.

[33] Heres A., Saldaña C., Toldrá F., Mora L. (2021). Identification of Dipeptides by MALDI-ToF Mass Spectrometry in Long-Processing Spanish Dry-Cured Ham. Food Chem. Mol. Sci..

[34] Sugimoto M., Sugawara T., Obiya S., Enomoto A., Kaneko M., Ota S., Soga T., Tomita M. (2020). Sensory Properties and Metabolomic Profiles of Dry-Cured Ham during the Ripening Process. Food Res. Int..

[35] Gallego M., Toldrá F., Mora L. (2022). Quantification and in Silico Analysis of Taste Dipeptides Generated during Dry-Cured Ham Processing. Food Chem..

[36] Zhu C.-Z., Tian W., Li M.-Y., Liu Y.-X., Zhao G.-M. (2017). Separation and Identification of Peptides from Dry-Cured Jinhua Ham. Int. J. Food Prop..

[37] Sforza S., Galaverna G., Schivazappa C., Marchelli R., Dossena A., Virgili R. (2006). Effect of Extended Aging of Parma Dry-Cured Ham on the Content of Oligopeptides and Free Amino Acids. J. Agric. Food Chem..

[38] Heres A., Yokoyama I., Gallego M., Toldrá F., Arihara K., Mora L. (2021). Antihypertensive Potential of Sweet Ala-Ala Dipeptide and Its Quantitation in Dry-Cured Ham at Different Processing Conditions. J. Funct. Foods.

[39] Marušić N., Aristoy M.-C., Toldrá F. (2013). Nutritional Pork Meat Compounds as Affected by Ham Dry-Curing. Meat Sci..

[40] Cho H.-B., Ahn J.-H., Yang H.-G., Lee J., Park W.-J., Kim Y.-W. (2021). Effects of PH and NaCl on Hydrolysis and Transpeptidation Activities of a Salt-Tolerant γ-Glutamyltranspeptidase from Bacillus Amyloliquefaciens S0904. Food Sci. Biotechnol..

[41] Fukao T., Suzuki H. (2021). Enzymatic Synthesis of γ-Glutamylvalylglycine Using Bacterial γ-Glutamyltranspeptidase. J. Agric. Food Chem..

[42] Hu X., Legler P.M., Khavrutskii I., Scorpio A., Compton J.R., Robertson K.L., Friedlander A.M., Wallqvist A. (2012). Probing the Donor and Acceptor Substrate Specificity of the γ-Glutamyl Transpeptidase. Biochemistry.

